# Analysis of blood lipid changes and influencing factors in physical examination population of a city in central China

**DOI:** 10.3389/fcvm.2022.996148

**Published:** 2022-11-08

**Authors:** Boya Zhu, Wenjing Wang, Mengying Li, Shuzhen Peng, Xiaodong Tan

**Affiliations:** ^1^School of Public Health, Wuhan University, Wuhan, China; ^2^School of Nursing, Hubei University of Chinese Medicine, Wuhan, China; ^3^Department of Physical Examination, Huangpi District People’s Hospital, Wuhan, China

**Keywords:** blood lipid, body mass index, elderly, cardiovascular disease, quantile regression

## Abstract

**Purpose:**

The prevalence of cardiovascular diseases (CVDs) associated with lipid levels is increasing worldwide. Our purpose is to analyze the distribution level and influencing factors of lipid in the whole population and to put forward suggestions for preventing abnormal lipid levels.

**Methods:**

The study was based on a sample of 91,480 Chinese who participated in a nationwide physical examination program in Wuhan, a midland city in China, in 2018. The distribution of blood lipid in the population was observed using average, and the relationship between the influencing factors and blood lipid level was observed by quantile regression (QR).

**Results:**

A total of 91,480 people were evaluated in this study, among which 59,165 (64.68%) were female with a mean age of 51.71 ± 10.82 years. QR results showed that different physical examination indexes had different effects on lipid levels. Fasting plasma glucose (FBG) has the largest QR coefficient and BMI had positive effects on total cholesterol (TC), triglyceride (TG), and low-density lipoprotein cholesterol (LDL-C). In males, age has a positive influence on TC, LDL-C, and high-density lipoprotein cholesterol (HDL-C), while in females, age has a positive influence on all four indexes.

**Conclusion:**

We found that the TC and LDL-C levels of females were more susceptible to age than males, and the lipid levels of older females were higher than males. BMI has a greater effect on lipid levels in males than in females. Regardless of gender should pay attention to dyslipidemia caused by diabetes and abnormal liver function.

## Introduction

Cardiovascular disease (CVDs) is one of the leading causes of morbidity and mortality worldwide, which is a disease of the heart and blood vessels, including coronary heart disease, cerebrovascular disease, rheumatic heart disease, and other diseases (1), and hyperlipidemia has been recognized as a major risk factor (2). Blood lipid is the general name of cholesterol, triglyceride (TG), and lipoprotein in serum, and these indicators are closely related to clinical symptoms (3, 4). Since elevated plasma cholesterol and triglyceride levels are associated with the development of atherosclerosis, particularly non–high–density lipoprotein cholesterol (non–HDL–C) and low-density lipoprotein cholesterol (LDL-C), lipid markers are generally considered strong indicators of CVDs damage (5). Results of a large population-based study show that elevated high-density lipoprotein cholesterol (HDL-C) is strongly associated with reduced atherosclerotic events (6). However, limited studies have assessed the relationship between lipid profile and mortality among the general population but few studies have been conducted in Chinese populations (7).

Alanine aminotransferase (ALT) is an enzyme mainly present in liver cells and usually present in serum at low levels (8). Glutamic oxalacetictransaminase (GOT) is often used as an auxiliary examination for myocardial infarction and myocarditis. However, ALT and COT levels are significantly elevated during hepatocyte injury and are sensitive and reliable markers of liver inflammation. Extensive epidemiological studies have shown that CVDs is associated with serum liver enzyme concentrations, in part due to underlying non-alcoholic fatty liver disease (NAFLD) and its consequent effects on metabolism (9). Several cohort studies showed that elevated ALT and GOT levels were associated with an excessive risk of all-cause and cardiovascular death, and the risk increased with age (10, 11). The relationship between liver serum markers and cardiovascular risk is unclear. In addition, age and obesity may influence the association with mortality. Substantial clinical and epidemiological evidence indicates that obesity can directly or indirectly increase the incidence and mortality of CVDs s, including coronary heart disease, heart failure, hypertension, stroke, atrial fibrillation, and sudden cardiac death (12). Metabolic syndrome (MS), a risk factor for CVDs, is associated with higher BMI and dyslipidemia, which are due to elevated triglycerides (TG) and reduced HDL-C. Overweight and obesity affect individuals of all ages, but are especially common among middle-aged adults and getting younger. Old age has long been considered a risk factor for CVDs, but there is now evidence that the incidence of CVDs in adults over the age of 50 is declining, and that CVDs is moving toward younger people (5).

The development of dyslipidemia is a continuous and long-term process, and the change in lipid index can reflect the progress of dyslipidemia to a certain extent. Therefore, it is of great significance to study the relationship between lipid index and influencing factors. At present, considering that few studies have discussed the general trend of blood lipid and the influence of other indicators on blood lipid in the population undergoing a physical examination. Therefore, through the physical examination program from Huangpi District, Wuhan, we analyzed the status of blood lipid examination among people of all ages, to explore the risk factors of blood lipid, analyze the degree of influence, and provide guidance for the prevention of CVDs. In the meanwhile, we adopted the method of quantile regression (QR) to explore the distribution of blood lipids in the population and the relationship between the influencing factors. Compared with ordinary regression, this kind of regression is more applicable and intuitive (13). In this case, our results will be closer to the actual situation and reflect different degrees of influence.

## Materials and methods

### Study participants

This cross-sectional study was initiated from the nationwide physical examination program in Huangpi District, Wuhan, to evaluate dynamic trends in TC, TG, LDL-C, and HDL-C levels with influencing factor in the general Chinese population. The nationwide physical examination is carried out according to the age group of the population. The children aged 0–6 are headed by the District Education Bureau and the Maternal and Child Health Hospital, the students aged 7–18 are headed by the District Education Bureau, and the residents aged 19 and above are organized by the streets.

In this study, we initial included 99,470 participants aged 3–99 years old in 2018. After excluding the population lacking fasting blood glucose, triglyceride and other data, a total of 91,480 people were evaluated were included in the study, with complete physical indicators. Participants in the study were voluntary and data confidentiality was guaranteed by the research team.

### Measurements

The items of examination include general basic examination, routine laboratory examination, laboratory biochemical examination, imaging examination, and screening for non-communicable diseases. In order to facilitate the physical examination of residents, Huangpi District government purchased professional mobile health service vehicles. The vehicle is equipped with automatic biochemical analyzer, portable color Doppler ultrasound diagnostic instrument, urine analyzer, portable 12—lead ECG machine, medical automatic sphygmomanometer, folding portable body height, weight and body temperature integrated machine, cardiovascular and cerebrovascular disease screening equipment, defibrillator and other equipment, which can carry out blood routine, urine routine, blood glucose and lipid, liver function, renal function and other test items. All examinations are conducted by trained doctors and nurses, strictly following the physical examination system.

### Statistical analysis

Data were analyzed with SPSS 25.0 and STATA 15.0. To describe our sample, general characteristics were described as mean values and standard deviations. The *t*-test was used for differences in general characteristics between groups; the chi-square test was used for categorical variables. We used QR to explore the risk factors associated with the distribution of four lipid markers (TC, TG, HDL, and LDL). We selected ten quantiles (from 10th to the 100th) of indicators based on the lowest to the highest indicators. These indicators were chosen as dependent variables since these indicators reflect the human body’s blood lipid situation and ensure a sufficient sample size. In the meanwhile, age, BMI, Fasting plasma glucose (FBG), ALT, and AST were chosen as a concomitant variable. QR coefficients were calculated and graphs were drawn to describe the influence of covariables on dependent variables more vividly. *p*-values of < 0.05 were considered statistically significant.

## Results

### Baseline characters

A total of 91,480 people were evaluated in this study, among which 59,165 (64.68%) were female with a mean age of 51.71 ± 10.82 years. Characteristics of the participants by gender are presented in Table 1. In this study, the six indexes of age, BMI, FBG, TG, ALT, and GOT were slightly lower in females than in males (*P*<0.005), while TC was higher than in males, with statistical significance.

**TABLE 1:** Descriptive characteristics of participants by gender.

Variable	Total (*n* = 91,480)	Male (*n* = 32,315)	Female (*n* = 59,165)	T	*P*-value
Age (year)	51.97 ± 11.13	52.43 ± 11.66	51.71 ± 10.82	9.300	<0.001
BMI (Kg/m^2^)	24.02 ± 3.47	24.29 ± 3.36	23.87 ± 3.52	17.328	<0.001
FBG (mmol/L)	5.35 ± 1.70	5.43 ± 1.80	5.30 ± 1.64	11.397	<0.001
TC (mmol/L)	4.77 ± 0.93	4.72 ± 0.92	4.80 ± 1.80	–12.591	0.003
TG (mmol/L)	1.56 ± 1.29	1.73 ± 1.59	1.46 ± 1.08	30.280	<0.001
LDL (mmol/L)	2.70 ± 0.80	2.70 ± 0.80	2.70 ± 0.80	–1.176	<0.001
HDL (mmol/L)	1.35 ± 0.35	1.28 ± 0.35	1.39 ± 0.34	–42.592	0.449
ALT (U/L)	20.05 ± 13.10	23.64 ± 14.72	18.09 ± 11.67	62.632	<0.001
GOT (U/L)	22.08 ± 9.14	23.79 ± 10.00	21.14 ± 8.50	42.328	<0.001
ALB (g/L)	46.14 ± 3.12	46.54 ± 3.29	45.92 ± 3.00	29.192	<0.001
Bil (μmol/L)	11.39 ± 5.31	12.90 ± 6.00	10.57 ± 4.69	64.785	<0.001
Scr (μmol/L)	65.68 ± 17.30	78.30 ± 16.55	58.79 ± 13.37	193.521	<0.001
BUN (mmol/L)	5.07 ± 1.52	5.42 ± 1.59	4.87 ± 1.45	52.698	<0.001

Descriptive characteristics of participants by gender [X ± SD]. X, mean; SD, standard deviations; BMI, body mass index; FBG, fasting plasma glucose; TC, total cholesterol; TG, triglycerides; LDL, low-density lipoprotein; HDL, high-density lipoprotein; ALT, alanine aminotransferase; GOT, glutamic oxalacetictransaminase; ALB, albumin; TBil, total bilirubin; Scr, serum creatinine; BUN, blood urea nitrogen.

**FIGURE 1:**
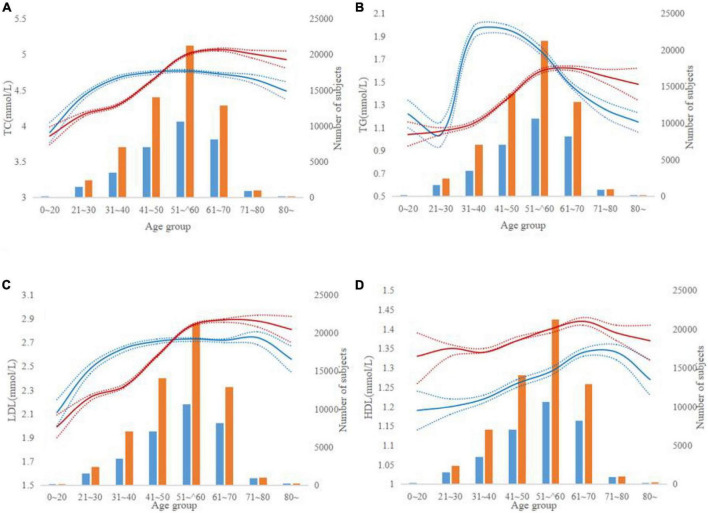
Lipid levels according to age groups. **(A–D)** The red and blue lines indicate trends for males and females, respectively. The dotted line represents a 95% confidence interval and the bars represent the number of males and females. **(A–D)** Represent TC, TG, LDL, and HDL levels by age groups.

**FIGURE 2:**
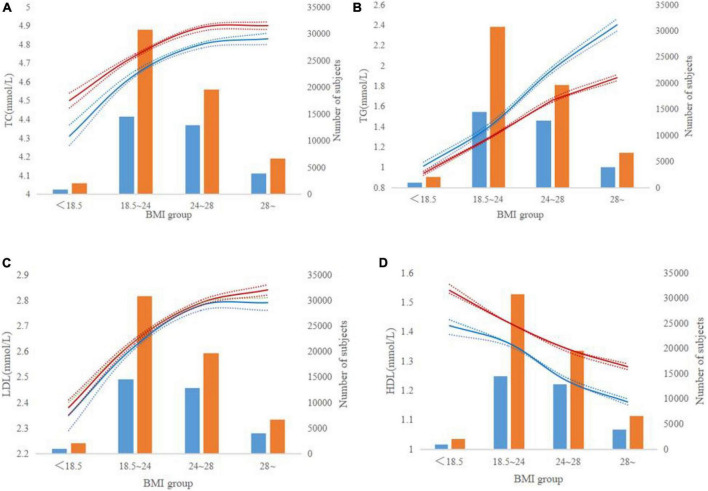
Lipid levels according to body mass index groups. **(A–D)** The red and blue lines indicate trends for males and females, respectively. The dotted line represents a 95% confidence interval and the bars represent the number of males and females. **(A–D)** Represent TC, TG, LDL, and HDL levels by BMI groups.

To assess the effects of age and BMI on lipid levels, we divided the whole population, calculated the average lipid levels of each population, and plotted them by sex (Figures 1, 2). Apparently, non-linear trends between age and lipid levels were found. For males, TC levels increased from 4.42 mmol/L in that 21∼30 years to 4.67 mmol/L in that 31–40 years, plateaued between 41 and 70, and then decreased from 4.49 mmol/L in those > 80 years (Figure 1A). For females, TC levels significantly increased from 4.29 mmol/L in that 31∼40 year to 5.07 mmol/L in that 61∼70 year, and then it stabilizes. The observed trends for TG in females differed from those in males (Figure 1B). TG levels significantly increased from 1.04 mmol/L in those 21∼30 years to 1.94 mmol/L in those 31∼40 years in males, but the increase was smaller for females. LDL levels increase rapidly in males younger than 50 years from 2.11 to 2.71 mmol/L and in females between 40 and 60 years from 2.59 to 2.84 mmol/L (Figure 1C). Thus, in those < 50 years, the males had higher LDL levels than females. However, females have higher LDL levels than males as they get older. When females are over 80, their LDL levels were 2.81 mmol/L, but for males, it is 2.56 mmol/L. HDL changes were similar in males and females, and HDL levels were consistently higher in females than in males (Figure 1D).

TC levels increased with the increase of BMI in both males and females and the female was high than the male (Figure 2A). TG levels significantly increased from 1.39 mmol/L in the normal population to 1.96 mmol/L in those overweight men (Figure 2B). In women, a similarly regular increasing trend of TG was found in those overweight, but the average TG was lower in women than in men. LDL levels were less than 2.4 mmol/L for both men and women who weighed less (<18.5 Kg/m^2^). When BMI increased by more than 24 Kg/m^2^, LDL increased by more than 2.78 mmol/L (Figure 2C). HDL values decreased with increasing BMI in the whole population (Figure 2D). In men, HDL levels decreased from 1.36 to 1.23 mmol/L, while BMI increased from 18.5 ∼24 to 24 ∼ 28 Kg/m^2^. For women, HDL levels decreased from 1.43 to 1.34 mmol/L, while BMI increased from 18.5 ∼24 to 24 ∼ 28 Kg/m^2^.

### Quantile regression analyses of lipid levels

**FIGURE 3:**
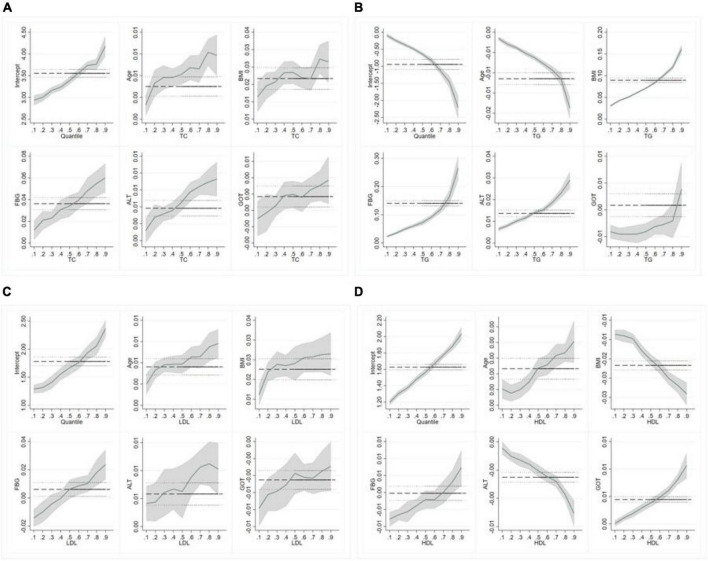
Quantile regression (QR) results for lipid levels (males). **(A–D)** Represent the quantile regression results of TC, TG, LDL-c, and HDL-c levels, respectively.

**FIGURE 4:**
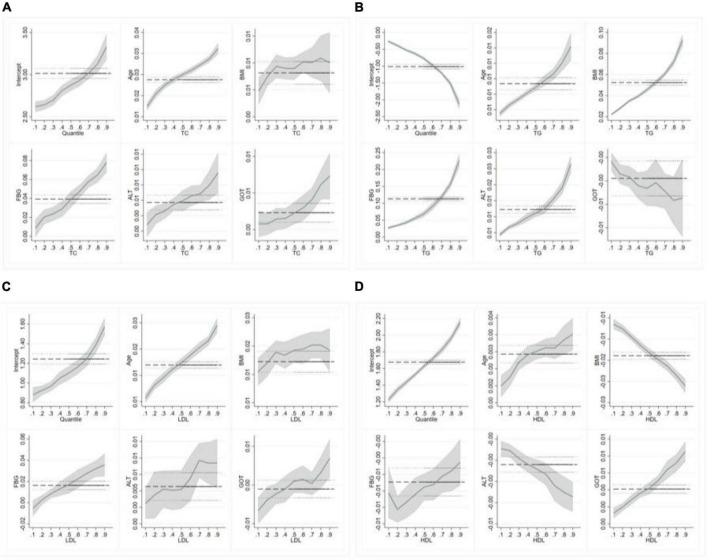
Quantile regression (QR) results for lipid levels (females). **(A–D)** Represent the quantile regression results of TC, TG, LDL-c, and HDL-c levels, respectively.

To further determine the potential influencing factors for the distribution of lipid levels, Tables 2, 3 and Figures 3, 4 list coefficients of multivariate QR between the lipid levels and their influencing factors for males and females independently. In males, FBG and ALT were positively associated with TC levels in all quantiles, and the coefficients of FBG and ALT showed an increasing trend with TC levels. The quantile coefficients of age, BMI and GOT increased first, then decreased (Q0.6), and then increased with the change of TC level, maximum in high quantiles (Q0.8). BMI, FBG, ALT and GOT were positively associated with TG levels in all quantiles. Interestingly, age is inversely proportional to TG levels. In the results of LDL, the highest QR coefficient of BMI was 0.026 (Q0.6∼Q0.9). The regression coefficients of age and ALT fluctuated but generally showed an upward trend. The regression coefficient of GOT increased gradually, but it was negatively correlated with LDL level. BMI and ALT were negatively correlated with LDL levels. Q0.7 is the zero bound point of the FBG quantile regression coefficient, the quantile regression coefficient is less than 0 in the low quantile and then gradually increases to 0.007 (Q0.9).

**TABLE 2:** Quantile regression coefficients and 95% confidence intervals between lipid levels and variables for males.

Variable	Q0.1	Q0.2	Q0.3	Q0.4	Q0.5	Q0.6	Q0.7	Q0.8	Q0.9
**TC**									
Age	*0.003 (0.002, 0.005)	*0.005 (0.004, 0.007)	*0.006 (0.005, 0.007)	*0.006 (0.005, 0.007)	*0.006 (0.005, 0.007)	*0.007 (0.006, 0.008)	*0.007 (0.005, 0.008)	*0.008 (0.007, 0.010)	*0.008 (0.006, 0.010)
BMI	*0.016 (0.012, 0.021)	*0.020 (0.015, 0.024)	*0.021 (0.017, 0.024)	*0.021 (0.017, 0.024)	*0.023 (0.020, 0.027)	*0.022 (0.020, 0.026)	*0.022 (0.017, 0.026)	*0.027 (0.022, 0.032)	*0.026 (0.020, 0.033)
FBG	*0.012 (0.003, 0.021)	*0.021 (0.011, 0.030)	*0.023 (0.016, 0.030)	*0.031 (0.023, 0.039)	*0.034 (0.025, 0.043)	*0.039 (0.032, 0.047)	*0.048 (0.037, 0.059)	*0.055 (0.045, 0.065)	*0.060 (0.047, 0.073)
ALT	*0.003 (0.002, 0.005)	*0.005 (0.003, 0.006)	*0.005 (0.004, 0.006)	*0.006 (0.004, 0.007)	*0.007 (0.005, 0.008)	*0.008 (0.006, 0.009)	*0.008 (0.007, 0.010)	*0.009 (0.007, 0.010)	*0.009 (0.008, 0.011)
GOT	−0.001 (−0.003, 0.001)	−0.0003 (−0.002, 0.002)	0.001 (−0.001, 0.002)	*0.002 (0.000, 0.004)	*0.002 (0.000, 0.004)	0.002 (0.000, 0.003)	*0.003 (0.000, 0.005)	*0.003 (0.001, 0.005)	*0.004 (0.001, 0.006)
**TG**									
Age	*−0.002 (−0.002, -0.001)	*−0.003 (−0.003, -0.002)	*−0.004 (−0.004, -0.003)	*−0.005 (−0.00, -0.005)	*−0.006 (−0.00, -0.005)	*−0.008 (−0.00, -0.007)	*−0.009 (−0.01, -0.008)	*−0.012 (−0.01, -0.010)	*−0.019 (−0.02, -0.016)
BMI	*0.030 (0.028, 0.0330	*0.043 (0.040, 0.045)	*0.050 (0.048, 0.053)	*0.061 (0.058, 0.064)	*0.071 (0.068, 0.075)	*0.084 (0.080, 0.088)	*0.101 (0.096, 0.106)	*0.119 (0.113, 0.125)	*0.162 (0.150, 0.173)
FBG	*0.023 (0.018, 0.028)	*0.035 (0.029, 0.040)	*0.049 (0.042, 0.056)	*0.061 (0.054, 00.067)	*0.073 (0.064, 0.081)	*0.092 (0.081, 0.102)	*0.118 (0.104, 0.132)	*0.162 (0.140, 0.184)	*0.266 (0.225, 0.306)
ALT	*0.006 (0.006, 0.007)	*0.008 (0.007, 0.009)	*0.010 (0.009, 0.011)	*0.012 (0.010, 0.013)	*0.014 (0.013, 0.015)	*0.015 (0.014, 0.017)	*0.019 (0.017, 0.020)	*0.023 (0.020, 0.026)	*0.029 (0.025, 0.033)
GOT	*−0.004 (−0.005, -0.003)	*−0.005 (−0.006, -0.004)	*−0.005 (−0.006, -0.003)	*−0.005 (−0.00, -0.003)	*−0.004 (−0.00, -0.003)	*−0.003 (−0.00, -0.002)	*−0.003 (−0.00, -0.001)	−0.002 (−0.005, 0.001)	0.004 (−0.002, 0.001)
**LDL**									
Age	*0.004 (0.003, 0.005)	*0.005 (0.004, 0.006)	*0.006 (0.005, 0.007)	*0.006 (0.005, 0.007)	*0.006 (0.005, 0.007)	*0.007 (0.006, 0.007)	*0.007 (0.005, 0.008)	*0.007 (0.006, 0.009)	*0.008 (0.006, 0.009)
BMI	*0.016 (0.013, 0.020)	*0.022 (0.019, 0.025)	*0.024 (0.020, 0.028)	*0.024 (0.021, 0.026)	*0.024 (0.020, 0.028)	*0.026 (0.022, 0.029)	*0.026 (0.022, 0.030)	*0.026 (0.022, 0.030)	*0.026 (0.021, 0.032)
FBG	*−0.014 (−0.021, -0.008)	*−0.001 (−0.017, -0.003)	−0.004 (−0.011, 0.003)	0.000 (−0.006, 0.005)	0.006 (0.005, 0.008)	*0.009 (0.002, 0.015)	*0.010 (0.003, 0.018)	*0.018 (0.009, 0.027)	*0.024 (0.015, 0.033)
ALT	*0.006 (0.004, 0.007)	*0.006 (0.005, 0.007)	*0.006 (0.005, 0.008)	*0.007 (0.006, 0.008)	*0.007 (0.005, 0.008)	*0.008 (0.007, 0.009)	*0.008 (0.007, 0.009)	*0.008 (0.007, 0.010)	*0.008 (0.006, 0.010)
GOT	*−0.008 (−0.010, -0.006)	*−0.006 (−0.008, -0.005)	*−0.006 (−0.008, -0.004)	*−0.005 (−0.007, -0.003)	*−0.004 (−0.00, -0.002)	*−0.004 (−0.00, -0.003)	*−0.004 (−0.00, -0.003)	*−0.003 (−0.010, -0.001)	*−0.003 (−0.005, 0.000)
**HDL**									
Age	*0.002 (0.002, 0.002)	*0.002 (0.002, 0.003)	*0.002 (0.002, 0.002)	*0.002 (0.002, 0.003)	*0.003 (0.002, 0.003)	*0.003 (0.002, 0.003)	*0.003 (0.003, 0.004)	*0.003 (0.003, 0.004)	*0.004 (0.003, 0.004)
BMI	*−0.014 (−0.015, -0.012)	*−0.014 (−0.015, -0.013)	*−0.015 (−0.016, -0.014)	*−0.018 (−0.019, -0.017)	*−0.020 (−0.022, -0.019)	*−0.022 (−0.02, -0.021)	*−0.025 (−0.02, -0.024)	*−0.027 (−0.02, -0.025)	*−0.029 (−0.032, -0.026)
FBG	*−0.008 (−0.011, -0.005)	*−0.007 (−0.008, -0.005)	*−0.006 (−0.008, -0.003)	*−0.004 (−0.006, -0.002)	−0.002 (−0.005, 0.001)	−0.002 (−0.005, 0.000)	0.000 (−0.003, 0.003)	0.003 (−0.001, 0.007)	*0.007 (0.002, 0.013)
ALT	0.000 (−0.001, 0.000)	*−0.001 (−0.001, -0.001)	*−0.001 (−0.002, -0.001)	*−0.002 (−0.002, -0.001)	*−0.002 (−0.002, -0.002)	*−0.002 (−0.003, -0.002)	*−0.003 (−0.00, -0.002)	*−0.004 (−0.00, -0.003)	*−0.005 (−0.00, -0.004)
GOT	0.000 (−0.001, 0.001)	*0.001 (0.001, 0.002)	*0.002 (0.001, 0.003)	*0.003 (0.002, 0.004)	*0.004 (0.003, 0.004)	*0.005 (0.004, 0.006)	*0.006 (0.005, 0.007)	*0.008 (0.007, 0.009)	*0.011 (0.009, 0.012)

*P < 0.05.

**TABLE 3:** Quantile regression coefficients and 95% confidence intervals between lipid levels and variables for females.

Variable	Q0.1	Q0.2	Q0.3	Q0.4	Q0.5	Q0.6	Q0.7	Q0.8	Q0.9
**TC**									
Age	*0.017 (0.016, 0.018)	*0.020 (0.020, 0.021)	*0.022 (0.022, 0.023)	*0.024 (0.023, 0.025)	*0.025 (0.024, 0.026)	*0.026 (0.025, 0.027)	*0.027 (0.026, 0.028)	*0.029 (0.028, 0.030)	*0.031 (0.020, 0.033)
BMI	*0.005 (0.002, 0.008)	*0.007 (0.005, 0.010)	*0.009 (0.007, 0.012)	*0.009 (0.007, 0.011)	*0.009 (0.006, 0.011)	*0.010 (0.008, 0.013)	*0.010 (0.007, 0.013)	*0.011 (0.007, 0.015)	*0.010 (0.005, 0.015)
FBG	*0.008 (0.000, 0.017)	*0.020 (0.014, 0.027)	*0.024 (0.017, 0.030)	*0.029 (0.021, 0.037)	*0.039 (0.032, 0.047)	*0.048 (0.042, 0.054)	*0.058 (0.050, 0.066)	*0.065 (0.055, 0.074)	*0.078 (0.066, 0.090)
ALT	*0.003 (0.001, 0.005)	*0.004 (0.003, 0.005)	*0.005 (0.003, 0.006)	*0.005 (0.004, 0.007)	*0.006 (0.004, 0.007)	*0.007 (0.005, 0.008)	*0.007 (0.005, 0.008)	*0.008 (0.006, 0.010)	*0.010 (0.007, 0.012)
GOT	0.001 (−0.001, 0.003)	0.001 (−0.001, 0.002)	0.001 (0.000, 0.003)	0.002 (0.000, 0.003)	*0.002 (0.000, 0.004)	*0.003 (0.001, 0.005)	*0.004 (0.002, 0.006)	*0.006 (0.004, 0.008)	*0.007 (0.004, 0.011)
**TG**									
Age	*0.005 (0.005, 0.006)	*0.007 (0.006, 0.007)	*0.007 (0.007, 0.008)	*0.008 (0.008, 0.009)	*0.009 (0.008, 0.009)	*0.010 (0.010, 0.010)	*0.011 (0.010, 0.011)	*0.012 (0.011, 0.013)	*0.014 (0.013, 0.016)
BMI	*0.022 (0.021, 0.024)	*0.028 (0.027, 0.030)	*0.035 (0.033, 0.037)	*0.039 (0.038, 0.041)	*0.046 (0.044, 0.048)	*0.052 (0.050, 0.055)	*0.061 (0.058, 0.063)	*0.072 (0.069, 0.075)	*0.093 (0.087, 0.098)
FBG	*0.027 (0.022, 0.031)	*0.034 (0.031, 0.037)	*0.042 (0.037, 0.047)	*0.055 (0.049, 0.060)	*0.068 (0.061, 0.075)	*0.091 (0.081, 0.100)	*0.118 (0.108, 0.129)	*0.156 (0.142, 0.171)	*0.227 (0.203, 0.250)
ALT	*0.004 (0.004, 0.005)	*0.007 (0.006, 0.007)	*0.008 (0.007, 0.009)	*0.010 (0.009, 0.011)	*0.011 (0.010, 0.012)	*0.012 (0.011, 0.014)	*0.015 (0.014, 0.017)	*0.019 (0.017, 0.021)	*0.026 (0.024, 0.029)
GOT	*−0.002 (−0.003, -0.002)	*−0.003 (−0.004, -0.003)	*−0.004 (−0.005, 0.003)	*−0.004 (−0.00, -0.003)	*−0.005 (−0.00, -0.003)	*−0.004 (−0.00, 0.003)	*−0.005 (−0.00, -0.003)	*−0.006 (−0.00, -0.004)	*−0.005 (−0.009, 0.002)
**LDL**									
Age	*0.011 (0.010, 0.011)	*0.013 (0.012, 0.014)	*0.015 (0.014, 0.015)	*0.016 (0.015, 0.017)	*0.017 (0.017, 0.018)	*0.019 (0.018, 0.020)	*0.020 (0.019, 0.021)	*0.022 (0.021, 0.022)	*0.025 (0.023, 0.026)
BMI	*0.015 (0.014, 0.018)	*0.017 (0.015, 0.019)	*0.019 (0.017, 0.021)	*0.018 (0.006, 0.021)	*0.019 (0.017, 0.21)	*0.019 (0.017, 0.022)	*0.020 (0.018, 0.023)	*0.020 (0.017, 0.023)	*0.019 (0.015, 0.023)
FBG	−0.005 (−0.013, 0.002)	0.002 (−0.003, 0.008)	*0.008 (0.004, 0.013)	*0.013 (0.008, 0.018)	*0.017 (0.011, 0.023)	*0.022 (0.016, 0.028)	*0.027 (0.022, 0.033)	*0.032 (0.026, 0.038)	*0.036 (0.027, 0.045)
ALT	*0.004 (0003, 0.006)	*0.005 (0.003, 0.006)	*0.005 (0.004, 0.006)	*0.005 (0.004, 0.006)	*0.005 (0.004, 0.006)	*0.006 (0.005, 0.007)	*0.007 (0.006, 0.008)	*0.007 (0.005, 0.008)	*0.007 (0.005, 0.009)
GOT	*−0.003 (−0.005, -0.002)	*−0.002 (−0.004, 0.000)	−0.001 (−0.003, 0.002)	−0.001 (−0.002, 0.001)	0.000 (−0.001, 0.002)	(−0.001, 0.002)	0.000 (−0.001, 0.002)	0.002 (−0.001, 0.004)	*0.003 (0.001, 0.006)
**HDL**									
Age	*0.002 (0.002, 0.002)	*0.002 (0.002, 0.003)	*0.003 (0.002, 0.003)	*0.003 (0.003, 0.003)	*0.003 (0.003, 0.003)	*0.003 (0.003, 0.003)	*0.003 (0.003, 0.004)	*0.003 (0.003, 0.004)	*0.004 (0.003, 0.004)
BMI	*−0.012 (−0.013, -0.010)	*−0.013 (−0.013, -0.012)	*−0.015 (−0.015, -0.014)	*−0.016 (−0.017, -0.016)	*−0.018 (−0.019, -0.017)	*−0.020 (−0.021, -0.019)	*−0.021 (−0.022, 0.020)	*−0.024 (−0.025, -0.022)	*−0.026 (−0.028, -0.025)
FBG	*−0.008 (−0.011, -0.006)	*−0.010 (−0.012, -0.009)	*−0.009 (−0.011, -0.007)	*−0.008 (−0.011, 0.006)	*−0.008 (−0.009, -0.006)	*−0.007 (−0.009, -0.005)	*−0.006 (−0.008, -0.004)	*−0.006 (−0.009, -0.003)	*−0.005 (−0.008, -0.001)
ALT	*−0.001 (−0.002, -0.001)	*−0.002 (−0.002, 0.001)	*−0.002 (−0.002, -0.002)	*−0.002 (−0.003, -0.002)	*−0.002 (−0.003, -0.002)	*−0.003 (−0.003, -0.002)	*−0.003 (−0.004, -0.003)	*−0.004 (−0.004, -0.003)	*−0.004 (−0.005, -0.003)
GOT	*0.001 (0.001, 0.002)	*0.002 (0.001, 0.003)	*0.003 (0.002, 0.003)	*0.004 (0.003, 0.004)	*0.004 (0.004, 0.005)	*0.005 (0.004, 0.006)	*0.006 (0.006, 0.007)	*0.007 (0.006, 0.008)	*0.009 (0.007, 0.010)

*P < 0.05.

In females, age, FBG, ALT and GOT were positively associated with TC levels in all quantiles, and they have the largest QR coefficient in high quantiles. The regression coefficients of BMI fluctuated slightly but generally showed an upward trend. Age, BMI, FBG, and ALT were positively associated with TG levels. The figure showed the specific information about the trend of covariates effects. The confidence interval starts very narrow and then gets wider, indicating a bigger standard error in high quantiles when analyzing the interaction between TG and GOT in females. In addition, the regression coefficients of BMI, ALT, GOT and LDL also fluctuated significantly, and the maximum regression coefficient appeared in Q.7∼Q0.8. In the results of HDL, age and GOT were positively associated with HDL levels, others are the opposite.

Comparing the QR coefficients for men and women, we found that women’s TC and LDL levels were more susceptible to age than men’s. What is more noteworthy is that age changes in males and females have opposite effects on TG values. BMI has a greater effect on lipid levels in men than in women, The regression coefficients of BMI, TC, and LDL increased rapidly at low quantile and then fluctuated little. FBG had the greatest effect on TC and TG in both men and women, especially in high quantiles. The effects of GOT on TC and LDL were also different in men and women.

## Discussion

We used a QR model to assess the distribution and influencing factors of lipids, including demographic characteristics, blood glucose, and liver serum markers. The results show that different factors have different influence trends and degrees on blood lipid. We found that Men’s blood lipid levels increase rapidly during middle age, while women’s increase with age. And the influence of age change on the TG value of men and women is the opposite in old. Men are more likely to be affected by BMI. The effects of FBG, Alt, and GOT on lipid levels should be paid more attention to in high quantile. These results will provide more detailed guidance for maintaining normal blood lipid levels and preventing CVDs.

### Age and body mass index effects on blood lipid

In our study, the effect of age on TC, HDL, and LDL was similar in men and women except for TG. With the increase of age, the three indexes of blood lipid, TC, HDL, and LDL, have different degrees of increase. We usually think of old age as a risk factor for CVDs, but some experiments have shown that it might be an attenuation of the association between total cholesterol (TC) and mortality with an increase in age (14). The mortality rate and levels of TC have a U-curve pattern, and the very low levels of TC are associated with increased frail syndrome and mortality. Similarly, a study based on older adults found that elevated TC levels were significantly associated with a reduced risk of all-cause mortality, primarily due to a reduced risk of non-CVDs (15). This suggests that for the very old, the causes of death are complex, not just lipid factors. At the same time, high TC levels gradually appeared younger. We not only need to pay attention to the lipid profile of young people but also need to pay attention to the health of the elderly from a comprehensive and multi-angle. In men, TG decreases with age, while in women it decreases. Sex differences in lipid metabolism are the result of sex chromosomes and sex-specific hormones. Women store more lipids and have a higher percentage of body fat than men, and their TG synthesis rate is higher than that of men (16, 17). Aging in men enhances the negative effects of androgens on blood lipids (18). Among the elderly, the levels of TC, TG, and LDL in women are significantly higher than those in men, which may be the reason why the onset of CVDs in women is later than that in men (19). In recent years, the number of CVDs cases and deaths has been higher in women than in men (20). With the increase in BMI, blood lipid levels also increased to different degrees. In our study, LDL significantly did not increase linearly with age, which is in contrast to a population study in the United States. In overweight people, with increasing BMI, the level of LDL-C tends to be flat or decreased in males (21).

### Fasting plasma glucose and liver serum markers effects blood lipid

Consistent with our findings, existing research demonstrated that hyperglycemia was in concert with the potential risk factors of CVDs s, for instance, elevated TG and TC (22, 23). People with high fasting glucose had a higher prevalence of elevated lipids (24). Elevated serum glucose levels can affect enzymes such as serum glutamic oxaloacetic aminotransferase (SGOT), ALT, alkaline phosphatase (ALP), and creatinine kinase (CK), thus affecting serum levels. The characteristics of dyslipidemia in diabetes mellitus are low HDL-C, increased triglyceride (TG), increased low-density lipoprotein (sdLDL), and postprandial lipidaemia (25). The relationship between diabetes and CVDs is complex, with studies suggesting that a high ratio of TC to HDL-C predicts type 2 diabetes. Therefore, it is necessary not only to control blood glucose but also to carry out multi-factor intervention on dyslipidemia and hypertension to prevent vascular complications in diabetic patients. ALT was positively correlated with TC, TG, and LDL in the whole population. The effect of GOT on blood lipids in men and women is inconsistent. Recently, there is limited evidence available suggesting TG and decreased HDL-C concentrations always appear in MS, which may be related to insulin resistance (26). In a study in Taiwan, abnormal AST and ALT levels were associated with uric acid, TG, fasting blood glucose levels, and males. Currently, liver function enzymes, ALT, and GOT are emerging biomarkers of CVDs risk (27). However, since the causes of CVDs are complex, the true impact of ALT on CVDs remains to be studied (10). Therefore, attention should be paid to liver and kidney functions when abnormal blood lipids exist to prevent the occurrence of complications.

### Strengths and limitations

Our study had several strengths and shortcomings. In previous studies, few of them applied QR to select variables associated with blood lipid, which ensured the strengths of avoiding collinearity and increasing the robustness. In our study, QR both performed well in providing variable selections and describing their effects on blood lipid in different sites. Unlike the OLS, which can merely describe the partial effects of independent variables made on dependent variables, the QR model gives an overall analysis of how those factors affect the blood lipid whatever distribution the data meet, more accurate and robust. In addition, the database provided large sample sizes with representativeness, covering all ages.

However, the limitations of our study should be noted. First of all, this study is a cross-sectional study, and the results of the data cannot determine the causal relationship. And the regression coefficient is statistically significant, but the value is small. Second, although the population in this study is expected to cover all age groups, most of them are middle-aged people and there are data on teenagers. Therefore, the influence of lipids on adolescents needs to be further analyzed and explored. Third, our study participants were limited to those who participates in a national health examination for health promotion screening and appeared to be slightly healthier than others, and thus, they may not have been representative of the general population. In addition, there are racial differences in serum cholesterol levels, but this was not explored in our study.

## Conclusion

In general, we constructed robust regression models to conclude that some demographic characteristics and physical indicators affected blood lipid levels to varying degrees, which could provide scientific guidelines for health. Gender, age, BMI, high blood glucose, and high serum glutamine have certain negative effects on lipid levels, which can increase lipid levels, but different indicators have different effects. Men should pay more attention to the influence of obesity on blood lipids than women, and women should pay more attention to dyslipidemia caused by advanced age. In the prevention of CVDs s, we need to pay attention to the occurrence of diabetes, abnormal liver and kidney function, and other complications caused by the above-mentioned indicators. There is no consensus on the effect of lipid levels in young people, so more attention should be paid to this issue in the future.

## Data availability statement

The raw data supporting the conclusions of this article will be made available by the authors, without undue reservation.

## Author contributions

BZ, WW, and ML were responsible for manuscript writing and data analysis. SP contributed to the acquisition and collation of data. XT reviewed the manuscript and provided a critical review. All authors provided substantial contributions toward the completion of this manuscript, gave final approval of the version to be published, and agreed to be accountable for all aspects of the work.

## References

[1] Roth GA Mensah GA Johnson CO Addolorato G Ammirati E Baddour LM Global burden of cardiovascular diseases and risk factors, 1990–2019: update from the GBD 2019 study. *J Am Coll Cardiol.* (2020). 76:2982–3021. 10.1016/j.jacc.2020.11.010 33309175 PMC7755038

[2] Zhu Y Lu J-M Yu Z-B Li D Wu M-Y Shen P Intra-individual variability of total cholesterol is associated with cardiovascular disease mortality: a cohort study. *Nutr Metab Cardiovasc Dis.* (2019) 29:1205–13. 31383502 10.1016/j.numecd.2019.07.007

[3] Collaboration PS. Blood cholesterol and vascular mortality by age, sex, and blood pressure: a meta-analysis of individual data from 61 prospective studies with 55 000 vascular deaths. *Lancet.* (2007) 370:1829–39. 10.1016/S0140-6736(07)61778-4 18061058

[4] Andersson C Vasan RS. Epidemiology of cardiovascular disease in young individuals. *Nat Rev Cardiol.* (2018) 15:230–40. 10.1038/nrcardio.2017.154 29022571

[5] Shin HJ McCullough PA. Focus on lipids: high-density lipoprotein cholesterol and its associated lipoproteins in cardiac and renal disease. *Nephron Clin Pract.* (2014) 127:158–64. 10.1159/000363552 25343842

[6] Kim MK Han K Kim H-S Park Y-M Kwon H-S Yoon K-H Cholesterol variability and the risk of mortality, myocardial infarction, and stroke: a nationwide population-based study. *Eur Heart J.* (2017) 38:3560–6. 10.1093/eurheartj/ehx585 29069458 PMC6251576

[7] Kim WR Flamm SL Di Bisceglie AM Bodenheimer HC Public Policy Committee of the American Association for the Study of Liver Disease. Serum activity of alanine aminotransferase (ALT) as an indicator of health and disease. *Hepatology.* (2008) 47:1363. 10.1002/hep.22109 18366115

[8] Harada PH Cook NR Cohen DE Paynter NP Rose L Ridker PM. Relation of alanine aminotransferase levels to cardiovascular events and statin efficacy. *Am J Cardiol.* (2016) 118:49–55. 27317931 10.1016/j.amjcard.2016.04.012

[9] Mahady SE Wong G Turner RM Mitchell P Macaskill P Craig JC Elevated liver enzymes and mortality in older individuals. *J Clin Gastroenterol.* (2017) 51:439–45. 10.1097/MCG.0000000000000622 27479143

[10] Afarideh M Aryan Z Ghajar A Noshad S Nakhjavani M Baber U Complex association of serum alanine aminotransferase with the risk of future cardiovascular disease in type 2 diabetes. *Atherosclerosis.* (2016) 254:42–51. 10.1016/j.atherosclerosis.2016.09.009 27684605

[11] Koliaki C Liatis S Kokkinos A. Obesity and cardiovascular disease: revisiting an old relationship. *Metabolism.* (2019) 92:98–107. 10.1016/j.metabol.2018.10.011 30399375

[12] Lin G He X Portnoy S. Quantile regression with doubly censored data. *Comput Stat Data Anal.* (2012) 56:797–812. 10.1111/j.1541-0420.2011.01667.x 21950348 PMC3312995

[13] Menotti A Puddu PE. Risk factors measured in middle-aged men predicting coronary events in geriatric age. *Int J Cardiol.* (2016) 222:1116–21. 10.1016/j.ijcard.2016.07.210 27545085

[14] Liang Y Vetrano DL Qiu C. Serum total cholesterol and risk of cardiovascular and non-cardiovascular mortality in old age: a population-based study. *BMC Geriatr.* (2017) 17:294. 10.1186/s12877-017-0685-z 29281976 PMC5745647

[15] Varlamov O Bethea CL Roberts CT Jr. Sex-specific differences in lipid and glucose metabolism. *Front Endocrinol.* (2015) 5:241. 10.3389/fendo.2014.00241 25646091 PMC4298229

[16] Li J Cao Y Xiao C. Subgroup analysis of the influence of body mass index on the association between serum lipids and cognitive function in Chinese population. *Lipids Health Dis.* (2019) 19:1. 10.1186/s12944-020-01314-7 32513187 PMC7282081

[17] Kolovou G Bilianou H Marvaki A Mikhailidis DP. Aging men and lipids. *Am J Mens Health.* (2011) 5:152–65. 10.1177/1557988310370360 20483870

[18] Karlson BW Palmer MK Nicholls SJ Barter PJ Lundman P. Effects of age, gender and statin dose on lipid levels: results from the VOYAGER meta-analysis database. *Atherosclerosis.* (2017) 265:54–9. 10.1016/j.atherosclerosis.2017.08.014 28863328

[19] Mozaffarian D Benjamin EJ Go AS Arnett DK Blaha MJ Cushman M Heart disease and stroke statistics—2016 update: a report from the American heart association. *Circulation.* (2016) 133:e38–360. 10.1161/CIR.0000000000000350 26673558

[20] Li H Ma J Zheng D Li X Guo X Wang J Sex differences in the non-linear association between BMI and LDL cholesterol in middle-aged and older adults: findings from two nationally representative surveys in China. *Lipids Health Dis.* (2021) 20:1–12. 10.1186/s12944-021-01591-w 34774059 PMC8590757

[21] Cui J Sun J Wang W Yasmeen N Ke M Xin H Triglycerides and total cholesterol concentrations in association with IFG/IGT in Chinese adults in Qingdao, China. *BMC Public Health.* (2018) 18:444. 10.1186/s12889-018-5286-z 29615002 PMC5883258

[22] Rhee EJ Han K Ko SH Ko KS Lee WY. Increased risk for diabetes development in subjects with large variation in total cholesterol levels in 2,827,950 Koreans: a nationwide population-based study. *PLoS One.* (2017) 12:e0176615. 10.1371/journal.pone.0176615 28545051 PMC5436642

[23] Kaur G Sudhera N Singh K Singh G Bassi DK. Effect of fasting blood glucose (FBG) on lipid metabolism and gender differences in the pattern of dyslipidemia in adults with type 2 diabetes in Northern India. *Stud Ethno Med.* (2017) 11:209–15.

[24] Suastika K Semadi I Dwipayana I Saraswati MR Gotera W Budhiarta A Dyslipidemia in diabetes: a population-based study in Bali. *Int J Gen Med.* (2019) 12:313–21. 10.2147/IJGM.S215548 31564954 PMC6730602

[25] Wu K-T Kuo P-L Su S-B Chen Y-Y Yeh M-L Huang C-I Nonalcoholic fatty liver disease severity is associated with the ratios of total cholesterol and triglycerides to high-density lipoprotein cholesterol. *J Clin Lipidol.* (2016) 10:420–5.e1. 10.1016/j.jacl.2015.12.026 27055973

[26] Hsieh M-H Lin W-Y Chien H-H Chien L-H Huang C-K Yang J-F Waist circumference, body mass index, serum uric acid, blood sugar, and triglyceride levels are important risk factors for abnormal liver function tests in the Taiwanese population. *Kaohsiung J Med Sci.* (2012) 28:470–6. 10.1016/j.kjms.2012.04.003 22974665 PMC11916595

[27] Weng SF Kai J Guha IN Qureshi N. The value of aspartate aminotransferase and alanine aminotransferase in cardiovascular disease risk assessment. *Open Heart.* (2015) 2:e000272. 10.1136/openhrt-2015-000272 26322236 PMC4548065

